# Acute Effects of Complex Hand Proprioceptive Task on Low-Frequency Hand Rest Tremor

**DOI:** 10.3390/s25206502

**Published:** 2025-10-21

**Authors:** Francesca Di Rocco, Emanuel Festino, Olga Papale, Marianna De Maio, Cristina Cortis, Andrea Fusco

**Affiliations:** 1 Department of Human Sciences, Society and Health, University of Cassino and Lazio Meridionale, 03043 Cassino, Italy; emanuel.festino@unicas.it (E.F.); olga.papale@unicas.it (O.P.); marianna.demaio@unicas.it (M.D.M.); 2 Department of Human Sciences and Promotion of Quality of Life, “San Raffaele” Open University of Rome, 00166 Rome, Italy; francesca.dirocco@uniroma5.it; 3 European University of Technology EUt+, 03043 Cassino, Italy; 4 Department of Medicine and Aging Sciences, University of “G. d’Annunzio” Chieti-Pescara, 66100 Chieti, Italy; andrea.fusco@unich.it

**Keywords:** neurophysiological monitoring, tremor, upper limb motor control, asymmetry, signal processing

## Abstract

Resting hand tremor is a low-frequency, involuntary oscillation influenced by mechanical and neural factors, often manifesting as inter-limb asymmetry. Therefore, the aim of this study was to investigate whether a single complex hand proprioceptive task can acutely modulate tremor in healthy young adults and whether it can induce asymmetry between limbs. Fifty participants (age: 25.0 ± 2.5 years) completed a 40-min proprioceptive task (anteroposterior, mediolateral, clockwise, and counterclockwise), with bilateral resting tremor recorded via triaxial accelerometry before and immediately after the intervention on both dominant and non-dominant limbs. Frequency-domain analysis showed a significant (*p* < 0.001) increase in tremor amplitude and a small decrease in mean frequency in the 2–4 Hz band immediately after the complex hand proprioceptive task for both limbs. These findings provide novel evidence that a single, wearable-based protocol can transiently modulate tremor dynamics, supporting the use of a non-invasive tool for neuromuscular monitoring in sport, rehabilitation, and clinical practice.

## 1. Introduction

Fine motor control is essential in both daily life and sport-specific tasks, particularly in disciplines requiring high levels of neuromuscular precision. Among the parameters used to assess motor variations, physiological tremor has emerged as a promising non-invasive biomarker of neuromuscular function, able to reflect the integration of central and peripheral mechanisms involved in motor execution. This involuntary low-amplitude oscillation offers valuable insights into neuromotor performance and asymmetry [1]. Physiological tremor originates from a complex combination of mechanical, reflexive, and central nervous system contributions, and can be modulated by different factors such as fatigue, proprioceptive demands, or training history [2]. Recent studies [3,4] have demonstrated that tremor amplitude is particularly sensitive to fatigue or postural perturbation, especially in low-frequency bands (2–4 Hz), suggesting a reduced sensorimotor stability and a greater recruitment of fast-twitch motor units.

In sport science, tremor analysis has gained relevance as a non-invasive method to monitor neuromuscular fatigue, with increased tremor power reported after strenuous military tasks [3]. This method has also been used to detect inter-limb asymmetries, with greater variability in tremor amplitude attributed to asymmetrical motor patterns, in elite dancers [5]. Although the literature [6,7,8] has mainly focused on physiological or postural tremor in clinical or dynamic settings, the acute modulation of resting tremor during static fine-motor conditions remains largely unexplored. Moreover, studies lack sensitive approaches able to capture micro-fluctuations or side-dependent changes in motor steadiness. Therefore, frequency-domain analyses of accelerometric tremor signals, such as log-transformed power spectral density (Log[PSD]) and mean frequency, have emerged as more sensitive and quantitative biomarkers of neuromuscular adaptations [1,3] to overcome these limitations.

In addition to analytical methods, complex hand proprioceptive tasks, which involve multi-joint stabilization with rich sensory feedback, could provide an ecologically valid approach to influencing sensorimotor control [9] and investigating tremor mechanism. While wobble board (WB) protocols are traditionally applied to the lower limb, their rationale extends to upper-limb fine-motor control, where proprioceptive/balance devices can improve postural control, joint stability, and neuromuscular coordination [10,11]. When combined with wearable inertial measurement units (IMUs) [12,13], these protocols could provide a portable and objective method for assessing fine motor control and hand–eye coordination [14] across diverse populations, such as healthy individuals and patients [15].

Although resting tremor has been extensively studied in clinical populations, its assessment in healthy individuals remains unexplored. Understanding acute tremor responses to complex exercise may provide valuable insights into neuromuscular fatigue and inter-limb asymmetries [16,17]. To the best of our knowledge, no studies have systematically investigated whether a complex hand proprioceptive task can elicit acute changes in resting tremor parameters. Therefore, the present study aimed to evaluate the acute effects of a complex hand proprioceptive task on the Log[PSD] and mean frequency of resting hand tremor, as well as inter-limb asymmetry, in healthy individuals. We hypothesized that the complex hand proprioceptive task would induce acute changes in both tremor amplitude and frequency components, reflecting short-term neuromuscular adaptations.

## 2. Materials and Methods

### 2.1. Study Design

In accordance with the Declaration of Helsinki, this within-subject, pre-post experimental study, was approved on 9 June 2021 by the Institutional Review Board of the Department of Human Sciences, Society, and Health of the University of Cassino and Lazio Meridionale (approval number 11748) to investigate the acute effects of a complex hand proprioceptive task on the inter-limb asymmetry and spectral amplitude of resting hand tremor in healthy individuals. All participants provided written informed consent after being fully briefed on the aims, procedures, and voluntary nature of their participation. They were explicitly informed that they could withdraw from the study at any time without any consequences.

Inter-limb asymmetry in tremor amplitude is commonly analyzed in clinical populations, such as individuals with essential tremor or Parkinson’s disease, and it is often used as a marker of neuromuscular imbalance. Previous studies [18,19] investigated long-term training effects (e.g., strength, yoga, or resistance-based programs over 4–6 weeks) on motor asymmetry. However, to the best of our knowledge, no prior studies have examined the acute effect of a proprioceptive task on inter-limb asymmetry and spectral amplitude of resting tremor. To address this gap, the present study introduced a novel methodological approach requiring multi-planar movement and engaging small intrinsic hand muscles. The complex hand proprioceptive task was designed to simulate dynamic upper-limb tasks using visual-motor feedback, similar to interactive environments such as exergaming or virtual tasks, that may increase adherence across diverse populations (e.g., athletes, sedentary adults, youth, and older individuals) [20,21]. Unlike traditional hand training protocols that often lack complexity in motor patterns, the proposed task challenged participants across multiple axes of motion, requiring rapid neuromuscular adjustments and fine motor corrections. We hypothesized that this short-duration, motorically demanding task would show measurable modifications in inter-limb asymmetry of tremor. Therefore, resting tremor assessments were performed before (PRE) and immediately after (POST) the complex hand proprioceptive task on both dominant and non-dominant hands, with a tri-axial accelerometry used to extract tremor-related PSD parameters. Based on previous studies [22,23,24], fine motor skill evaluations were scheduled in a single session during the mid-morning hours to align with the body’s natural peak in alertness and motor coordination, thereby providing a more accurate assessment of an individual’s fine motor capabilities.

### 2.2. Participants

Fifty healthy young adults, recruited among the student population of the University of Cassino and Lazio Meridionale, voluntarily participated in the study. They were selected as a representative sample due to their homogeneity in terms of age, health status, and physical activity level, reducing potential confounding variables [1].

Participants were excluded if they reported pre-existing diagnosed conditions such as neurological conditions, cardiovascular, respiratory, and/or metabolic diseases, hypertension, osteoporosis, musculoskeletal injury of the back or lower extremities that had occurred during the past year, visual and vestibular disorders, or any drug use. Complying with the Regulation (EU) 2016/679 of the European Parliament and Council of 27 April 2016, known as the General Data Protection Regulation (GDPR), which protects individuals’ rights and privacy regarding personal data processing, to ensure anonymity, each participant was given a unique identification code, and personal data were used for statistical analysis purposes only.

### 2.3. Procedures

Before starting the official testing procedures, all participants were familiarized with the experimental protocols. A dedicated evaluator ensured that the testing environment met the following conditions: room temperature between 20 and 23 °C controlled by the air-conditioning system [25]; number of individuals in the room limited to essential personnel only; standardized lighting and room layout; all the testing equipment (WB, TPDev-IMU, and computer screen) properly set up; and consistent instructions provided to all participants. To minimize the influence of individual habits on resting hand tremor, participants were instructed to refrain from moderate-to-vigorous physical activity and to abstain from alcohol consumption and caffeine intake for at least 24 h prior to the experimental sessions [26]. Moreover, all assessments were scheduled in the mid-morning (between 9:00 and 13:00) to minimize the potential influence of circadian fluctuations. The total time for data collection was on average approximately 1 h per participant.

#### 2.3.1. Anthropometric Measurements

Anthropometrical parameters, including height (in meters) and body mass (in kilograms), were collected for each participant. The measurements were obtained using a scale with an integrated stadiometer (Seca, model 709; Vogel & Halke, Hamburg, Germany), which has an accuracy of 0.1 kg for body mass and 0.1 cm for height. The body mass index (BMI) was calculated as the weight (kg) divided by the height squared (m^2^). Dominant hand was determined by asking “Which hand do you write with?” [27]. Participants’ characteristics are presented in Table 1.

#### 2.3.2. Hand Rest Tremor Measurement

A tri-axial accelerometer (TalentPlayers^®^ TPDev-IMU, firmware version 1.3, Palermo, Italy) was used to evaluate the hand rest tremor. The TPDev-IMU is a small wearable electronic device integrating a 6 degree-of-freedom microelectromechanical system inertial sensor, able to provide acceleration, in meter on second squared (m/s^2^), and rotational data, in degree on second (deg/s), along the 3 orthogonal axes (X, Y, and Z), with a sampling frequency of 100 Hz. Data are analyzed in real-time by the device, using proprietary mathematical models taking into account the cinematic, dynamic, and energetic features of the motion [28].

Participants were seated upright on a chair with the back supported, feet flat on the floor. During the assessment, the tested limb was positioned with the elbow flexed at ~90°, forearm pronated, and hand resting on the lap. The TPDev-IMU was fixed under the palm of the middle finger level and attached to the hand using a common elastic band, with the contralateral limb resting on the armrest. This position was used across all participants to ensure consistency of signal acquisition and to minimize voluntary muscular activity that could interfere with the accuracy of resting tremor recordings, as detailed in a previous study [1]. Recordings were taken for 15 s by a stopwatch (Garmin International, Kansas City, MO, USA). Hand resting tremor was recorded for the dominant and non-dominant limb PRE and POST complex hand proprioceptive tasks as described in Section 2.3.3.

#### 2.3.3. Complex Hand Proprioceptive Task

The complex hand proprioceptive task was performed using a WB (WSP, Well Sport Project, G.S.J. Services S.r.l., Rome, Italy) integrating a triaxial accelerometer (Phidget Spatial 0/0/3 Basic 1041, Phidgets Inc., Calgary, AB, Canada). The WB provides real-time visual feedback through a custom software interface connected to a high-resolution monitor [29]. Participants controlled the WB’s tilt using one hand by moving the motion marker displayed on the monitor. The dynamic task required keeping the motion marker in the moving target zone in four different conditions that followed different movement trajectories: clockwise, counterclockwise, anteroposterior, and mediolateral. In the anteroposterior and mediolateral conditions, the speed of the target zone was adaptively modulated in real-time based on task performance, following a vertical (sagittal plane) and horizontal (frontal plane) trajectory, respectively. Conversely, in the clockwise and counterclockwise conditions, the speed of the target zone was fixed, following a predeterminate circular trajectory, independent of participant performance.

Following a standardized 1-min familiarization, each participant completed one 15-s trial per movement pattern and limb (eight trials total) in randomized order with 30 s of rest between trials. The total duration of the complex hand proprioceptive task was approximately 30 min.

#### 2.3.4. Frequency Analysis

The acceleration signal was sampled at a frequency of 100 Hz using the TPDev-IMU. Raw tri-axial acceleration signals (X, Y, Z axes, in m/s^2^) were collected during each 15-s hand resting tremor assessment and exported into Microsoft Excel (version 2017, Microsoft Corp., Redmond, WA, USA) only for preliminary organization and labeling. For each participant, limb (dominant vs. non-dominant), and condition (PRE vs. POST), the total acceleration magnitude (||*a*||) [30,31,32] was calculated using the square root of the sum of squares of the three axes, according to the equation:

$$\left\|a\right\|=\sqrt{a_{x}^{2}+a_{y}^{2}+a_{z}^{2}}$$

For each participant, the initial 5 s of each trial were excluded from analysis to mitigate transient adaptation effects, thus minimizing mechanical artifacts and enhancing the reliability of the subsequent analyses [3]. Therefore, in line with previous studies on hand resting and physiological tremor [3,5], only the final 10 s of data were retained for analysis, providing 1000 samples (frequency resolution of 0.1 Hz).

Before frequency-domain decomposition, the signal was demeaned to remove direct current offsets. Two complementary filters were then applied in a dual-branch design: a second-order zero-phase Butterworth Low-Pass filter (LP) with 10 Hz cutoff, and a corresponding High-Pass filter (HP) with 10 Hz cutoff [30,31,32]. The LP branch preserved low-frequency components for analysis of the 2–4 Hz range, while the HP branch isolated higher-frequency components for the analysis of the 10–20 Hz range. This approach ensured attenuation of undesired frequency content without phase distortion or artificial leakage between bands.

For each condition and participant, the PSD was estimated using Welch’s method with a Hamming window covering the entire 10-s segment (length equal to the available data samples for each trial; window overlap set to zero) [33], as implemented in MATLAB R2025a (MathWorks Inc., Natick, MA, USA). From the PSD function, two main indices describing the amplitude and frequency characteristics of the tremor signal were calculated for each waveform and experimental condition:

− The logarithmic amplitude indicator, *L*(*f*_1_, *f*_2_), was computed as the mean of the natural logarithm of the PSD values within each predefined frequency band of interest (2–4 Hz and 10–20 Hz), as described by Gajewski and colleagues [34]:

$$L\left(f_{1},\;f_{2}\right)=\frac{1}{{|f}_{2}-f_{1}|}\int_{f1}^{f_{2}}\ln[PSD\left(f\right)]df$$

− The mean frequency *F*(*f*_1_, *f*_2_) was calculated as the mean frequency weighted by the power components within each band:

$$F\left(f_{1},\;f_{2}\right)=\frac{\int_{f_{1}}^{f_{2}}f\cdot PSD\left(f\right)df}{\int_{f_{1}}^{f_{2}}PSD\left(f\right)df}[Hz]$$

Two frequency ranges were analyzed to capture distinct upper limb tremor components. The 2–4 Hz band reflects slow oscillations primarily associated with peripheral/mechanical and reflex contribution, whereas the 10–20 Hz band corresponds to the classical physiological tremor domain linked to central neural oscillation and motor-unit synchronization. According to recommendations for physiological tremor analysis, a natural logarithmic transformation was applied to all amplitude and power variables derived from the PSD. This transformation normalizes the spectral data, enhancing the interpretability and statistical robustness of subsequent analyses. The resulting indices L and F were extracted for both the 2–4 Hz and 10–20 Hz frequency bands, facilitating the quantification and comparison of tremor characteristics across experimental conditions.

**Figure 1:**
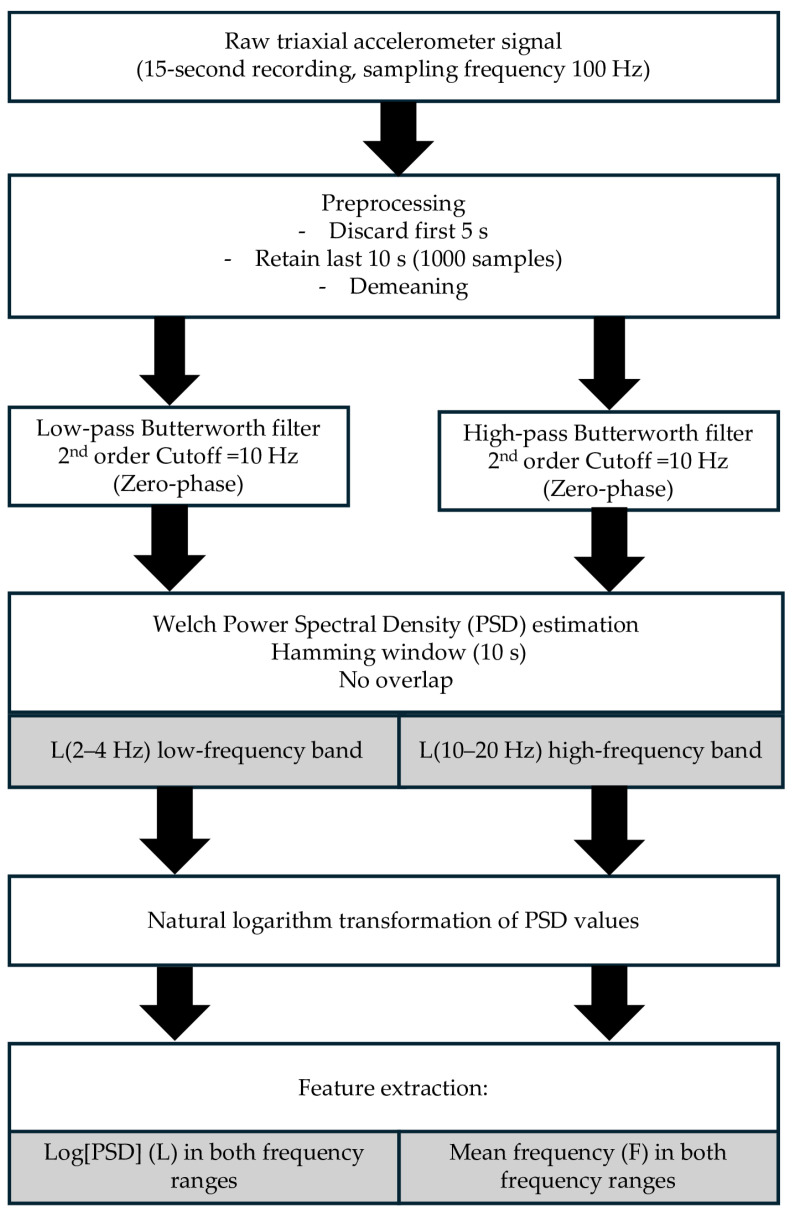
Signal processing diagram for tremor analysis. Black arrows indicate the sequential flow of data processing, from raw signal acquisition to feature extraction. White boxes represent the main processing steps (e.g., acquisition, preprocessing, filtering, spectral transformation), while gray boxes indicate the derived data outputs and computed features (e.g., frequency bands and extracted parameters).

All signal processing and frequency-domain analyses were conducted using custom routines implemented in MATLAB R2025a. The entire pipeline used to extract hand tremor characteristics from the raw signal accelerometer (Figure 1), including filtering, spectral decomposition, feature extraction, and data aggregation, was standardized and automated to ensure reproducibility and comparability across participants and conditions. This frequency-domain approach allowed for precise quantification of hand resting tremor amplitude components relevant to neuromuscular control and asymmetry, in line with recent findings on elite athletes and proprioceptive perturbation paradigms [5].

#### 2.3.5. Statistical Analysis

All statistical procedures were performed using MATLAB R2025a. The Shapiro–Wilk test was used to assess the normal distribution of the data. Descriptive statistics were computed for all variables and reported as means ± standard deviation (SD).

A repeated-measures analysis of variance (ANOVA) was conducted separately for each frequency band, with time (PRE and POST) and limb (dominant and non-dominant) as within-subject factors. The Greenhouse–Geisser correction was applied where the assumption of sphericity was violated. The level of statistical significance was set at *p* < 0.05 for the main effects and interactions, and *p* < 0.008 for Bonferroni-adjusted pairwise comparisons used to detect significant changes across condition and to control for Type I error. Effect sizes were calculated using partial eta squared (*η*_p_^2^) and interpreted according to Cohen’s guidelines: 0.01 ≤ *η*_p_^2^ < 0.06 a small effect, 0.06 ≤ *η*_p_^2^ < 0.14 a medium effect, and *η*_p_^2^ ≥ 0.14 a large effect [35].

## 3. Results

Descriptive statistics for the Log[PSD] and mean frequency values in the L2–4 and L10–20 frequency bands are reported in Table 2.

**Figure 2:**
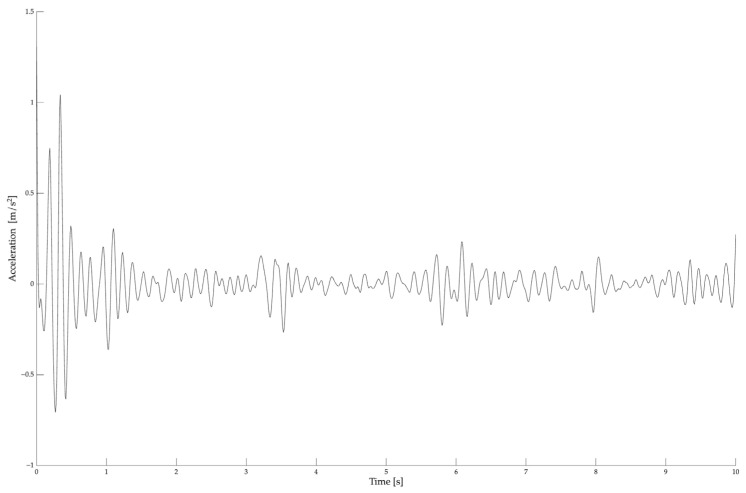
A raw acceleration signal of hand tremor registered for a representative participant before the complex hand proprioceptive task with the dominant limb.

**Figure 3:**
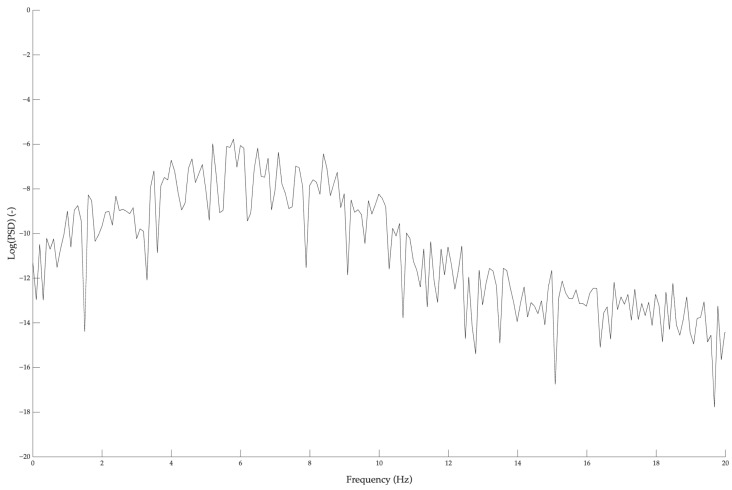
Power spectral density plot on a logarithmic scale (Log[PSD]) for a representative participant (the same whose raw acceleration course is presented in Figure 2). The graph shows the distribution of signal power across frequencies, with the logarithmic scale highlighting variations over a wide dynamic range.

Figure 2 presents a 10-s raw acceleration signal for a representative participant, while Figure 3 shows the power density function corresponding to the 10-s acceleration course, part of which is presented in Figure 2.

Repeated-measures ANOVA showed a significant main effect of testing time on Log[PSD] values in the L2–4 band (*F*_(1, 49)_ = 2939.07, *p* < 0.0001, *η*_p_^2^ = 0.983), with higher resting tremor amplitudes in the POST conditions. There was no significant main effect of limb (*F*_(1, 49)_ = 0.62, *p* = 0.43, *η*_p_^2^ = 0.012) nor a significant testing time x limb interaction (*F*_(1, 49)_ = 0.19, *p* = 0.66, *η*_p_^2^ = 0.004) in this frequency range. Bonferroni-corrected post hoc comparisons confirmed a significant increase in Log[PSD] values POST complex hand proprioceptive task protocol for both the dominant (PRE: −8.63 ± 1.95; POST: −4.84 ± 1.46; *p* < 0.001) and non-dominant (PRE: −8.69 ± 0.77; POST: −5.03 ± 1.34; *p* < 0.001) limbs. No significant difference was observed between limbs within the same testing time point (PRE: *p* = 1.000; POST: *p* = 1.000).

In the L10–20 frequency band, a significant main effect of testing time (*F*_(1, 49)_ = 86.56, *p* < 0.001, *η*_p_^2^ = 0.639), with slightly higher Log[PSD] values, was observed after the intervention. Bonferroni-corrected post hoc pairwise comparisons showed testing time differences for both dominant (PRE: −8.85 ± 1.64; POST: −7.84 ± 0.75; *p* < 0.001) and non-dominant (PRE: −9.10 ± 0.94; POST: −7.72 ± 0.85; *p* < 0.001) limbs. No significant main effect of limb (*F*_(1, 49)_ = 0.12, *p* = 0.72, *η*_p_^2^ = 0.003) nor testing time x limb interaction (*F*_(1, 49)_ = 1.94, *p* = 0.17, *η*_p_^2^ = 0.038) was found in this band.

The analysis of the mean frequency values showed a significant main effect of testing time in the 2–4 Hz band (*F*_(1, 49)_ = 36045.78, *p* < 0.0001, η_p_^2^ = 0.998), with lower mean frequency values observed in the POST condition. No significant main effect of limb (*F*_(1, 49)_ = 0.01, *p* = 0.92, η_p_^2^ < 0.001) emerged, while the testing time × limb interaction approached significance (*F*_(1, 49_) = 3.55, *p* = 0.066, η_p_^2^= 0.068). Post hoc tests confirmed significant reductions in mean frequency after the intervention for both dominant (PRE: 3.18 ± 0.19 Hz; POST: 2.72 ± 0.20 Hz; *p* < 0.001) and non-dominant (PRE: 3.15 ± 0.17 Hz; POST: 2.77 ± 0.18 Hz; *p* < 0.001) limbs.

In the 10–20 Hz frequency band, there was a marginal main effect of time (*F*_(1, 49)_ = 3.91, *p* = 0.054, η_p_^2^ = 0.074), although Bonferroni-corrected post hoc comparisons did not show significant differences between PRE and POST for either limb (all *p* > 0.05). Moreover, no significant main effect of limb (*F*_(1, 49)_ = 0.06, *p* = 0.81, η_p_^2^= 0.001) nor testing time × limb interaction (*F*_(1, 49)_ = 0.03, *p* = 0.86, η_p_^2^ < 0.001) was found.

## 4. Discussion

The present study investigated the acute effects of a complex hand proprioceptive task on the spectral amplitude and frequency of resting hand tremor in healthy adults. By using high-resolution accelerometric recordings and frequency-domain analyses, we aimed to verify whether an acute proprioceptive challenge could modulate tremor features and highlight potential inter-limb asymmetries. The main findings indicate a robust increase in tremor amplitude within the 2–4 Hz frequency band after the intervention with a modest reduction in mean frequency. These changes occurred independently of limb dominance, with no significant side differences or interaction effects. Moreover, a significant, although less pronounced, increase in tremor amplitude was observed in the higher-frequency band (10–20 Hz), while no significant changes in mean frequency emerged.

The observed increase in low-frequency tremor amplitude following a single session of complex hand proprioceptive task is consistent with previous evidence that proprioceptive or fatiguing interventions can acutely impact motor steadiness, especially in the lower-frequency spectral domain [1,5,36]. Similarly, Kuliś and colleagues [36] reported heightened tremor amplitude in speed skaters post-exercise, with pronounced changes in both low- and high-frequency bands. Although their work focused on the lower limbs and postural tremor, the underlying principle of neuromuscular adaptation is similar. Consistent with these findings, Papale and colleagues [1] reported that a WB hand exercise protocol led to acute changes in tremor and reduced inter-limb asymmetry, particularly during challenging motor tasks. The acute increase in tremor amplitude, most robust in the 2–4 Hz band although also evident in the 10–20 Hz, is likely attributable to transient neuromuscular adaptations following the proprioceptive challenge. This frequency range has been linked to central and spinal oscillatory processes, stretch reflex loops, and sensorimotor integration [36], describing low-frequency tremor as reflecting the dynamic interplay of mechanical and neural factors, with amplitude susceptible to acute modulation by fatigue, load, and sensory input. The present results extend this framework to fine motor control of the hand, supporting the hypothesis that the instability elicited by a complex hand proprioceptive task can acutely recalibrate the gain of sensorimotor circuits, leading to increased tremor output at rest.

Contrary to previous studies in elite athletes [5,36], our results did not show significant inter-limb differences in tremor amplitude or frequency, either before or after the intervention. A previous study [1] noted greater asymmetry in baseline tremor among healthy adults, which tended to normalize after exercise. However, our participants showed symmetrical motor profiles, probably because the sample did not include clinical populations or elite athletes engaging in chronic lateralized training, such as tennis or fencing. The lack of interaction effects between time and limb suggests that the acute neuromuscular adaptations elicited by complex hand proprioceptive tasks are generalizable across both dominant and non-dominant hands. This finding aligns with the literature [36], wherein observed symmetrical neuromuscular adaptation in the lower limbs have been reported following intense training in speed skaters. The potential reduction in inter-limb asymmetry through task-specific interventions represents a promising strategy for performance improvements and rehabilitation purposes.

Together with changes in amplitude, we observed a modest but statistically significant reduction in mean tremor frequency within the 2–4 Hz band after the exercise. This finding is in line with evidence [34,37,38] that neuromuscular fatigue and proprioceptive perturbation can shift tremor frequency toward lower ranges (e.g., 2–4 Hz), potentially reflecting increased reliance on slower, larger motor units or altered synchronization patterns. The observed shift in frequency could reflect the combined effects of the central and peripheral fatigue, including reduced central drive, altered corticospinal coherence, and temporary recalibration of spinal reflexes [36]. No significant changes in mean frequency were detected in the 10–20 Hz band, further supporting the interpretation that acute proprioceptive challenges mainly affect slower oscillatory components of motor output, with higher-frequency bands being more resilient and linked to intrinsic muscle and mechanical properties.

The present study’s methodology, involving tri-axial accelerometry and advanced spectral analysis, aligns with recent [39,40] best practices for quantifying tremor and motor micro-fluctuations in both clinical and performance settings. The use of log-transformed PSD metrics normalizes the distribution, improving statistical sensitivity and facilitating comparisons with previous findings. Our study focused on resting hand tremor, expanding upon studies [41,42] that have primarily examined postural or action tremor in sport and rehabilitation. Compared to a previous study [1] exploring inter-limb asymmetry using both clinical and functional tests, our study further characterizes the spectral features of tremor in response to a proprioceptive intervention. Although no reductions in asymmetry were observed in the present study (probably due to the healthy baseline of participants), the amplitude increases and frequency shifts are in line with findings from lower-limb studies in speed skaters [36] and military personnel [3].

Our findings support the utility of complex hand proprioceptive task protocols and frequency-domain tremor analysis for the acute assessment of neuromuscular state. The increase in low-frequency tremor amplitude after the exercise may represent as a sensitive biomarker of transient neuromuscular engagement and fatigue, complementing more traditional indicators such as electromyography (EMG). The lack of inter-limb differences highlights the generalizability of this response in healthy young adults, although further research is needed in athletic or clinical populations with established lateralized motor patterns. The non-invasiveness and portability of accelerometric assessment represents a valid opportunity for real-time monitoring in both laboratory and field settings. Moreover, the ability to quantify adaptations in tremor characteristics following brief interventions has the potential for optimizing training load, detecting early signs of maladaptation, and tailoring rehabilitation protocols.

An important feature of the present approach is its simplicity. In fact, a brief 15-s tremor recording analyzed with wearable sensors was sufficient to detect significant modulation following proprioceptive stimulation. This practical and time-efficient setup enhances the translational potential of the method, making it suitable for clinical screening, rehabilitation settings, and performance monitoring in sport and exercise contexts, where quick and reliable assessments are essential. Despite these meaningful findings, some limitations need to be addressed. The present study focused on a single, acute complex hand proprioceptive task intervention in healthy, non-elite young adults. Therefore, generalization to other populations, repeated training exposures, or clinical contexts should be approached cautiously. While alcohol assumption, caffeine intake, and engagement in moderate-to-vigorous physical activity were controlled during the 24 h before the experimental session, other potential confounders, such as sleep quality, stress, anxiety, and emotional or contextual factors that may influence tremor were not systematically assessed. These factors should be addressed in future studies, particularly considering recent evidence linking lifestyle behaviors, such as diet and sleep quality, in healthy student populations [43,44]. In addition, although the 10 s stationary window at 100 Hz provided 0.1 Hz spectral resolution and ~20 bins within the 2–4 Hz range, enabling robust estimation of band averaged Log[PSD] for within-subject PRE–POST comparisons, the very large effect sizes observed in this band (n_p2_ > 0.98) may also reflect the short recording duration. Longer recording durations (30–60 s) or multiple repeated segments would further stabilize low-frequency estimates and should be considered in future research. The sample size, while comparable to previous studies, may limit the detection of more specific effects or interactions. Thus, future studies should expand this approach to diverse cohorts, including older adults, athletes from various disciplines, or individuals with neuromotor disorders. Moreover, longitudinal designs and integration with complementary measures (e.g., EMG, neuroimaging, or kinematic data) would further clarify the mechanisms underlying tremor modulation and its practical applications. Finally, although simple IIR filters ensured stable preprocessing for short windows, future studies may consider higher-order Butterworth or FIR designs to further reduce phase distortion and improve spectral accuracy. Continued refinement of spectral analysis techniques could therefore enhance the sensitivity and interpretability of tremor as a biomarker for neuromuscular function.

## 5. Conclusions

The present study demonstrates that a single session of a complex hand proprioceptive task acutely increases resting hand tremor amplitude and shifts the mean frequency domain lower in healthy adults, without inducing significant inter-limb asymmetry. According to these findings, low-frequency tremor metrics are sensitive to proprioceptive exercises and can be used as non-invasive markers of acute neuromuscular adaptation. From an applied perspective, since generalizability across devices and sensor placements is a critical factor in activity recognition algorithms [13], ensuring robustness across measurement conditions is fundamental for translating tremor-based metrics into real-world monitoring. Therefore, complex hand proprioceptive protocols may represent a promising tool for monitoring and enhancing neuromotor performance in sport, rehabilitation, and clinical practice. In conclusion, although exploratory, this study shows the validity of short wearable-based assessments for capturing acute proprioceptive modulation of tremors. The simple and efficient approach represents a key strength, which supports its potential as a reliable biomarker tool in both clinical applications and performance monitoring.

## Figures and Tables

**Table 1:** Means and standard deviations of anthropometric characteristics of participants.

	Male *n* = 29	Female *n* = 21	Total *n* = 50
Age (years)	24.3 ± 1.1	26.1 ± 3.5	25.0 ± 2.5
Height (m)	1.7 ± 0.1	1.6 ± 0.1	1.7 ± 0.1
Weight (kg)	74.3 ± 9.7	56.6 ± 8.9	66.6 ± 13.0
BMI (kg/m^2^)	24.5 ± 2.2	21.6 ± 2.5	23.2 ± 2.7

*n* = number; BMI = Body Mass Index.

**Table 2:** Mean and standard deviation of hand resting tremor logarithmic amplitude indicators (L) and (F) before (PRE) and after (POST) the complex hand proprioceptive task.

Participants (*n* = 50)	PRE Dominant	POST Dominant	PRE Non-Dominant	POST Non-Dominant
L(2–4)	−8.63 ± 1.95	−4.84 ± 1.46 *	−8.69 ± 0.77	−5.03 ± 1.34 *
F(2–4) [Hz]	3.18 ± 0.20	2.72 ± 0.20 *	3.15 ± 0.17	2.77 ± 0.18 *
L(10–20)	−8.85 ± 1.64	−7.84 ± 0.75 *	−9.10 ± 0.94	−7.72 ± 0.85 *
F(10–20) [Hz]	14.61 ± 1.21	14.16 ± 1.05	14.31 ± 1.39	14.09 ± 1.17

*n* = number; * Indicate significantly difference from PRE within each limb (*p* < 0.001).

## Data Availability

Data available in a publicly accessible repository. The original data presented in the study are openly available in GitHub (GitHub Inc., San Francisco, CA, USA) at https://github.com/ccortis/DataTremor2.git (accessed on 2 September 2025).

## Abbreviations

The following abbreviations are used in this manuscript:

PSD	Power Spectral Density
Log[PSD]	Log-transformed Spectral Density
WB	Wobble Board
IMUs	Inertial Measurement Units
PRE	Before
POST	After
BMI	Body Mass Index
HP	High-Pass Single-Pole IIR Filter
LP	Low-Pass Single-Pole IIR Filter
ANOVA	Analysis of Variance
FIR	Finite Impulse Response
EMG	Electromyography
